# MR Elastography Using the Gravitational Transducer

**DOI:** 10.3390/s24248038

**Published:** 2024-12-17

**Authors:** Omar Isam Darwish, Vitali Koch, Thomas J. Vogl, Marcos Wolf, Katharina Schregel, Arnie Purushotham, Valérie Vilgrain, Valérie Paradis, Radhouene Neji, Ralph Sinkus

**Affiliations:** 1 Research Department of Imaging Physics and Engineering, School of Biomedical Engineering and Imaging Sciences, King’s College London, London WC2R 2LS, UK; radhouene.neji@kcl.ac.uk (R.N.);; 2 MR Predevelopment, Siemens Healthineers, 91052 Erlangen, Germany; 3 Department of Diagnostic and Interventional Radiology, University Hospital Frankfurt, 60629 Frankfurt am Main, Germany; 4 Centre for Medical Physics and Biomedical Engineering, Medical University of Vienna, 1090 Vienna, Austria; 5 Department of Neuroradiology, Heidelberg University Hospital, 69120 Heidelberg, Germany; 6 School of Cancer & Pharmaceutical Sciences, King’s College London, London WC2R 2LS, UK; 7 INSERM, Centre de Recherche sur l’Inflammation, Universite Paris Cite, 45018 Paris, France; 8 Department of Radiology, Hospital Beaujon, 92110 Clichy, France; 9 Department of Pathology, Hospital Beaujon, 92110 Clichy, France; 10 Laboratory for Vascular Translational Science (LVTS), INSERM U1148, 75877 Paris, France

**Keywords:** MR elastography, elastography, liver, MALFD, biomechanics, engineering

## Abstract

MR elastography is a non-invasive imaging technique that provides quantitative maps of tissue biomechanical properties, i.e., elasticity and viscosity. Currently, hepatic MR elastography is deployed in the clinic to assess liver fibrosis in MAFLD patients. In addition, research has demonstrated MR elastography’s ability to non-invasively assess chronic liver disease and to characterize breast cancer lesions and brain tumors. MR elastography requires efficient mechanical wave generation and penetration, motion-sensitized MRI sequences, and MR elastography inversion algorithms to retrieve the biomechanical properties of the tissue. MR elastography promises to enable non-invasive and versatile assessment of tissue, leading to better diagnosis and staging of several clinical conditions.

## 1. Introduction

Biomechanical integrity resides at the heart of the body’s homeostasis. Many pathologies manifest themselves by impacting tissue stiffness, such as chronic liver diseases, which cause inflammatory and fibrotic processes that generate the so-called “stiff liver”. For a long time, liver palpation has been used to estimate liver size and to feel for liver stiffness and eventually local masses to find signs of liver disease. The development of ultrasound elastography (USE) [1] and magnetic resonance elastography (MRE) [2,3] enhanced this radically by providing virtual palpation through quantitative maps of tissue stiffness and even tissue viscosity when conducted in 3D [4]. MR elastography has demonstrated its ability to non-invasively assess chronic liver disease [5,6] and to characterize breast cancer lesions [4,7] and brain tumors [8,9]. Currently, hepatic MRE is the most applied elastography technique in the clinic, where it is used to quantify liver fibrosis by measuring the shear stiffness [10] or the shear wave speed [6]. In addition, it has shown promise in grading liver inflammation by measuring the loss modulus [6].

**Figure 1:**
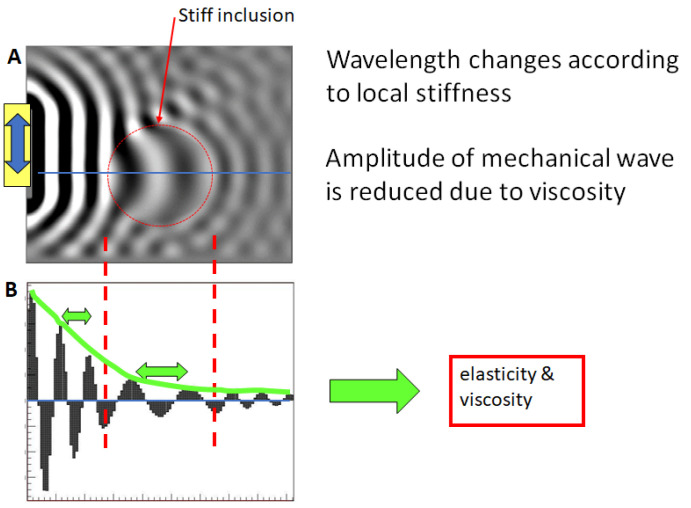
Dependence of shear wavelength on local stiffness. (**A**) Finite element simulation of a wavefield with a homogeneous background and a hard inclusion. (**B**) The corresponding line profile shows that the local wavelength (green arrows) changes depending on the underlying stiffness. Additionally, the amplitude of the wave drops due to an intrinsic loss mechanism (viscosity). “Imaging” the shear wave allows the local biomechanical properties to be recovered in return.

In general, elastography utilizes the intricate link between the properties of propagating mechanical shear waves, i.e., their amplitude, phase, and local wavelength, to uncover the underlying mechanical properties of tissue. Importantly, soft tissue is quasi-incompressible since it consists of 70% water, sometimes even more. Thus, the typical physician’s notion of tissue being “more or less compressible” is based on wrong terminology: tissue can be sheared to a certain extent given an exerted force, but it does not change its volume as water is incompressible. The spatially varying constitutive material properties result in the shear waves propagating faster or slower depending on whether the tissue is locally stiff or soft, respectively (Figure 1). For a fixed vibrational frequency, this translates into a longer or a shorter wavelength, which can be imaged via phase contrast-based magnetic resonance imaging (MRI) sequences [3,4,11,12].

Overall, MRE necessitates three essential steps: (I) efficient wave generation in a patient-friendly way, (II) wave image acquisition using phase-locked and motion-sensitized MRI sequences that yield high spatial fidelity, and (III) reconstruction of the underlying biomechanics in the most unbiased fashion.

## 2. Shear Wave Generation: MR Elastography Transducer

The generation of mono-frequent shear waves with sufficient amplitude and phase stability within the MRI environment is a nontrivial task. To date, several MRE transducer concepts have been proposed [13] with very different driving approaches: (I) current-driven electromagnetic coils that oscillate due to the Lorentz force in the main B0-field [4,14,15]; (II) a pneumatic approach connecting an active driver with a membrane via a flexible tube [16]; (III) a pulse density-modulated approach using compressed air [17]; and (IV) the gravitational transducer concept [18]. For MRE to yield high-quality maps of biomechanical properties, the transducer needs to transmit a pure frequency spectrum. Otherwise, parasite frequencies (upper harmonics) have a particular impact on the quality of the viscous biomechanical properties [19]. Each transducer concept has pros and cons, which relate to the flexibility, strength, pureness of the frequency spectrum, and ability to transmit waves, even under the application of an external mechanical load, e.g., the transducer positioned at the back of the patient.

**Figure 2:**
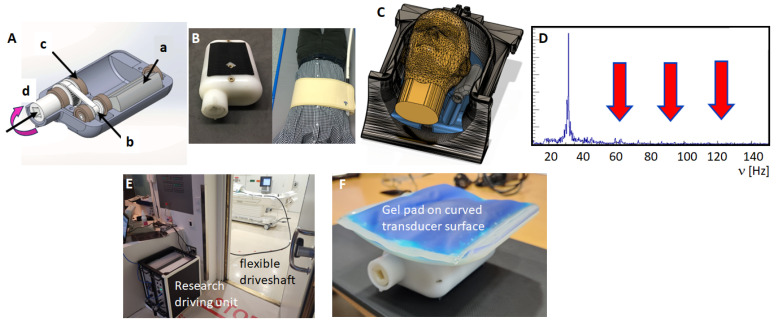
Gravitational transducer concept. (**A**) The gravitational transducer consists of a casing that hosts a spinning eccentric mass (a) which is connected via a gearbox (b,c) to an external flexible driveshaft (d). (**B**) The closed transducer is very compact, and for abdominal applications, it is strapped to the patient’s body via an elastic belt. (**C**) Its generic design allows seamless integration into concepts that enable, for instance, cranial MRE. (**D**) The frequency response spectrum when operated at 30 Hz shows no upper harmonics. (**E**) The research demonstrator version of the gravitational MRE concept had the driving unit outside the MRI room with the flexible driveshaft going through the waveguide towards the patient table. The picture shows the installation at the University Hospital Frankfurt am Main, Hesse, Germany. (**F**) To improve patient comfort for abdominal applications, the transducer has a curved contact plate with a gel pad. Curvature and size of the contact plate are easily adaptable to different applications.

Here, we focus on the description of the gravitational transducer approach and discuss its properties. The concept of the gravitational transducer utilizes the generic equivalence of acceleration with the force of a rotating eccentric mass for the generation of vibrations, which are similar to those found in a cell phone vibration motor. Figure 2A shows a sketch of the transducer design: the eccentric mass (a) is connected via a gearbox (b,c) to an external flexible driveshaft (d). The presence of the gearbox allows a reduction in the friction on the external driveshaft. The concept of a rotating eccentric mass has the advantage that the generated force grows quadratically with the increasing frequency, which leads to a vibrational amplitude independent of frequency [18]. This is in stark contrast to acoustically driven (pneumatic) approaches which always experience a reduction in amplitude with increasing frequency. The compact design of the transducer enables various abdominal or cerebral applications (Figure 2B,C) as the mass rotates independently regardless of whether any external load is applied to the casing of the transducer.

Typically, the loss in amplitude for acoustically driven approaches is compensated by an increase in driving power, which leads in turn to a nonlinear response of the system, i.e., the presence of upper harmonics. This leads to a degradation of data quality. The gravitational approach—in contrast—represents a linear system with a frequency response spectrum devoid of any upper harmonics (Figure 2D). Its concept, developed within an EU-funded Horizon2020 project (FORCE) at King’s College London, St Thomas’ Hospital, had the driving unit outside the MRI scanning room with the flexible driveshaft reaching through the waveguide and finally connected to the transducer at the patient’s bed (Figure 2E). To ensure patient comfort, the transducer has a curved contact plate cushioned with a gel pad. The gel pad allows compressional waves to pass without any attenuation into the patient’s body, while shear vibrations of the transducer are dampened, which increases patient comfort. This flexible design allows easy adaptions to other organs, such as the kidney, breast, and brain.

## 3. MR Elastography Sequence

**Figure 3:**
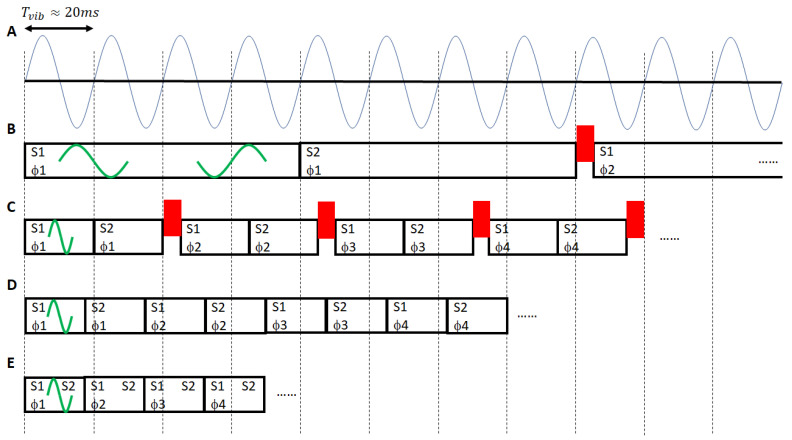
MRE sequence concepts. (**A**) Sinusoidal mechanical vibration generated by the MRE transducer is for clinical applications typically in the 40–60 Hz range. Thus, one period corresponds to roughly Tvib~20 ms. (**B**) SE-based sequences typically use MEGs (green) that operate at the vibration frequency. This leads to a long shot duration, i.e., the time interval encompassing excitation and readout. Temporal delays (red rectangle) are used to shift to the next wave phase Φ once all slices Si have been acquired (i ∈ [1, 2, 3 … N], N = # of slices). (**C**) Fractional motion encoding concepts enable significant scan time reduction at the cost of a loss to motion sensitivity. Initial approaches still had the temporal delays separate from each shot, thereby perturbing eddy current steady state [12]. (**D**) More sophisticated concepts overcame this by incorporating the delays into each shot, thereby further reducing scan time. (**E**) SMS finally opened a straightforward way to acquire 3D MRE datasets within a single breath hold.

MRE and diffusion-weighted imaging (DWI) sequences share many common properties: the intention of both is to quantify the motion of water molecules using motion-sensitized sequences. The fundamental difference between both concepts is that in diffusion we do not know when the individual water molecules are moving in time due to the Brownian motion. Hence, DWI is based on the concept of signal destruction without any impact on the net phase of the MR image [20]. In MRE, however, time is controlled via the vibration of the mechanical transducer. Hence, it is possible via a phase-locked approach to visualize the micron-level vibrations within the phase of the MR image [4,14]. Many phase-contrast MRI sequences can be used for MRE; however, gradient-echo (GRE) and spin-echo echo-planar imaging (SE-EPI) sequences are the ones most frequently employed. The fundamental concept of the phase locking is shown in Figure 3. The frequency of the motion encoding gradient (MEG) should be identical with the mechanical vibration frequency to obtain the largest sensitivity to motion. At 60 Hz, this leads to very long echo times, which require the utilization of spin-echo (SE) sequences to recover a sufficient signal-to-noise ratio (SNR), especially when operating at 3T. Consequently, a single SE-based excitation readout block spawns over approximately 50–66 ms (Figure 3B). Temporal delays (red blocks in Figure 3B) allow a shift in the MR acquisition relative to the mechanical vibration, providing images of the wave propagation at another timepoint (wave phase) throughout the oscillatory period of one mechanical vibration. Typically, four to eight of such wave phases equally spaced over one oscillatory cycle are acquired to later recover—during the MRE reconstruction process—the complex-valued displacement field. The long echo time (TE) results in prohibitively long acquisition times, which can be counterbalanced via echo-planar imaging (EPI) readout approaches. EPI leads, depending on echo spacing, phase field of view (FOV), and off-resonance, to geometrical distortions, which in turn will impact the recovery of the biomechanical properties, because higher-order spatial derivatives of the wavefields are necessary for solving the wave equation. GRE-based sequences using motion encoding gradients (MEGs) at the mechanical vibration frequency are one way to shorten acquisition times [21]. However, the long echo time of ~20 ms results in very poor SNR. The use of fractional MEGs is overcoming this limitation through sacrificing sensitivity to motion by shortening the MEG, which thus does not operate anymore at the mechanical vibrational frequency [11]. The resulting loss in the phase-to-noise ratio for wave propagation imaging can be partially recovered by more advanced motion encoding concepts such as Hadamard encoding [22]. The initial approaches used excitation readout blocks with durations that were still integer multiples of one mechanical oscillation period (Figure 3C) [23]. More advanced approaches incorporated the temporal delays into the duration of each individual shot, leading to a further shortening of the scan time (Figure 3D) [12]. Now, simultaneous multi-slice (SMS) excitation [24] has opened the gateway of shortening acquisition times even more, thereby enabling the capture of 3D datasets within one single breath hold (Figure 3E) [25]. Spiral readout concepts have also been proposed for brain MRE [26], including self-navigation for motion correction. Here, again, due to the long readout, B0-inhomogeneities and eddy current effects need to be properly compensated to avoid any impact on the fidelity of space.

## 4. MR Elastography Reconstruction

**Figure 4:**
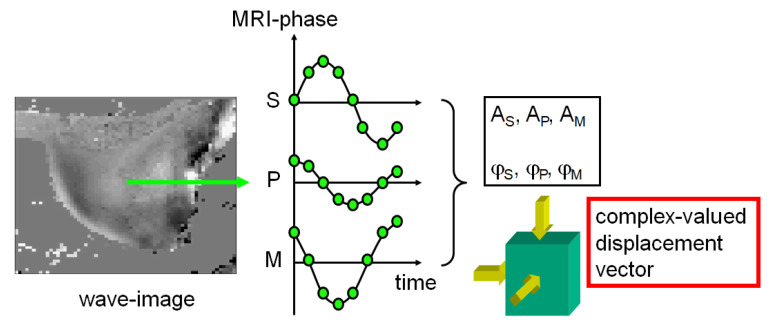
From MRE raw data to wavefield displacement vector. The MRE data acquisition provides snapshots of the propagating wave. When looked at pixel-wise, a sinusoidal modulation of the MRI phase is observed for each of the three encoding directions (readout [M], phase encoding [P], slice direction [S]). A temporal Fourier transform yields the corresponding amplitudes $A_{i}$ and phases $\varphi_{j}$ of the complex-valued displacement vector of the wavefield in direction i ∈ (M, P, S). This approach assumes a temporal steady state, i.e., there are no transient effects, and the time component is purely described by sinusoidal temporal modulation at the driving frequency.

The MRE sequence provides snapshots of the wavefield at different timepoints throughout an oscillatory cycle (Figure 4). Thus, when presented as a function of wave phase, each encoding direction will show a sinusoidal temporal MRI phase modulation for each single pixel. A temporal Fourier transform then yields the corresponding amplitudes Ai and phases ji of the wavefield, which constitute the steady state solution of the problem.

**Figure 5:**
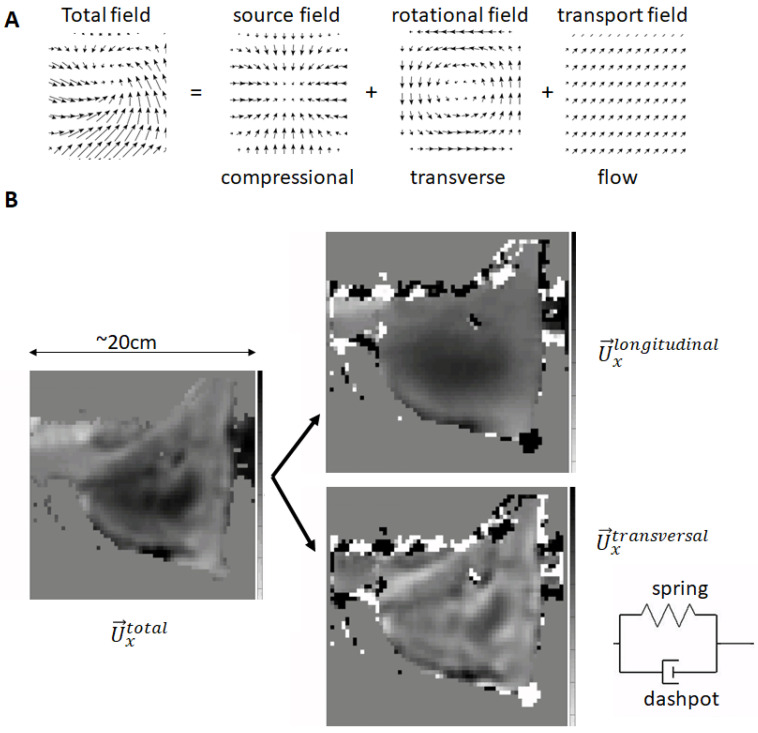
Mathematical foundations of MRE reconstruction. (**A**) In general, any wavefield is the sum of three fields that exhibit very different mathematical properties: a source field, a rotational field, and a transport field. In our case, the total displacement vector is the sum of the compressional (source) and the transverse (rotational, shear) wave, because there are no transport effects in our experiments. (**B**) Both waves exhibit very different mathematical properties and different wavelengths since they are coupled to different mechanical properties of tissue; here, as shown for in vivo data in breast tissue: the compressional wave in our frequency domain has a very long wavelength (~m) as it is linked to the bulk modulus. Remember that tissue is incompressible, leading to a bulk modulus of the order of GPa. Conversely, the shear wave is relatively short (~m) as it is linked to the shear modulus (~kPa). Bear in mind that both moduli differ by 6 orders of magnitude! The final solution of the complex-valued shear modulus can now be interpreted in many ways: one possibility is to view tissue as if shear stiffness (elasticity, spring) and shear loss (viscosity, dashpot) were organized as spring and dashpot connected in a parallel fashion (Voigt model). In reality, tissue exhibits a more complex mechanical response function leading to fractal-like mathematical representations due to its hierarchical organization.

The wave propagation is in general a 3D problem and can only be simplified to 2D or even 1D under specific boundary conditions [1]. The beauty of the MRE approach is that the measured displacement field constitutes the underlying wave solution of the problem at hand. Certainly, boundary conditions do impact the details of the wavefield due to reflections and scattering. Since the underlying physics, except under specific quantum mechanical conditions [27], is local, all we need to do is to invert the 3D wave propagation equation [28]. Mathematically, any continuous vector field can be decomposed into three different components which carry very different mathematical properties: (I) one field that has sources (curl-free), (II) one field—similar to the magnetic field— that has no start and no end (divergence-free), and (III) one that relates to transport effects (curl- and divergence-free, Figure 5A) [29]. In our case, due to the absence of any transport effects (there is no flow of tissue present in the data), the total displacement field is the sum of the compressional and the transverse (shear) wavefield (Figure 5B). These two fields probe very different properties of tissue: the compressional wavefield is linked to the bulk modulus of the tissue and propagates at the speed of sound in water, i.e., at 1550 m/s. Hence, it exhibits very long wavelengths, which are around ~30 m, at frequencies of around 50 Hz. This is mathematically very challenging to handle given the typical SNR in MRE data. Conversely, the transverse (shear) wave travels at speeds of around 1–10 m/s, resulting in wavelengths of around 2–20 cm! Given the typical pixel sizes of ~3–4 mm, such wavelengths can properly be resolved and used to calculate spatial derivatives of a higher order. For a correct inversion of the wave equation, it is necessary to first remove the compressional field. Using for instance its mathematical properties of “carrying” the sources of the mechanical wavefield, it is possible to mathematically remove it via the so-called “curl-operator”. Certainly, this is at the cost of an additional spatial derivative [30]. Other approaches try to remove the compressional component via high-pass filters in the Fourier domain [31] or via integration [32]. A combination of both utilizes divergence-free basis functions in the context of finite element modeling [19]. Once the compressional field has been removed, the remaining equation basically expresses Hooke’s Law: stress and strain are related via the shear modulus [28]. The exquisiteness in MRE is that the approach used to solve for the complex shear modulus is independent of any assumptions regarding the underlying rheological properties of tissue [30,33]. The single most important assumption is that the material is isotropic and linear. Thus, the shear modulus (elasticity) and loss modulus (viscosity) of the complex-valued shear modulus can be interpreted *a posteriori* in terms of the Voigt model, which assumes that tissue behaves as a spring and a dashpot in parallel (Figure 5B). In reality, tissue exhibits far more complex dispersion (i.e., frequency-dependent) properties which are likely to carry valuable diagnostic information [34,35].

It is important to stress that both elasticity and viscosity relate to the solid shear properties of the material, and the loss describes the ability of the material to extract energy from the propagating wave. The origin of this energy loss can be true absorption, i.e., conversion to heat or scattering, leading to a redistribution of the wave’s energy in space. Scattering-induced effects within tissue impact the frequency dependence of tissue and lead to an intricate mixture of constitutive and apparent effects governing the dispersion properties [36].

**Figure 6:**
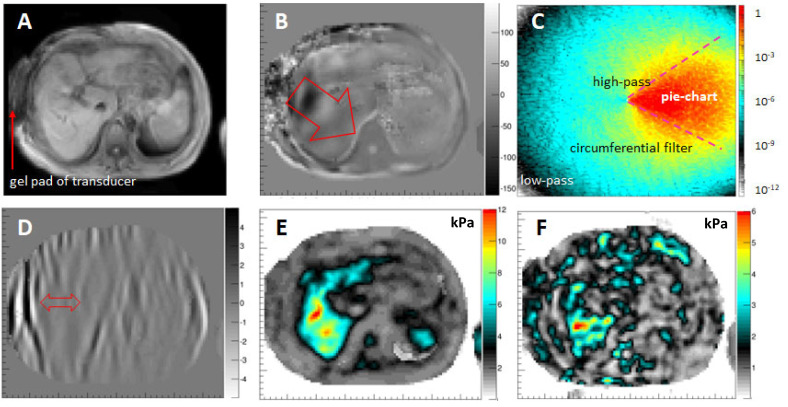
Directional wave filtering for 2D MRE and viscosity. (**A**) Magnitude image of the liver in transverse orientation. The gravitational liver transducer is located on the RHS of the patient with the gel pad visible (arrow). (**B**) The pattern of the wavefield in through-slice direction shows mainly a plane wave propagating towards the center of the patient (arrow, [mm]). (**C**) Amplitude of the Fourier transform of the wave image shown in B segmented in a pie chart fashion with additional low-pass, high-pass, and circumferential filters to generate a virtual plane shear wave in image-space after inverse Fourier transform. Bear in mind that the Z-scale is logarithmic. (**D**) Corresponding plane shear wave image showing longer wavelengths within the liver when compared to regions of subcutaneous fat (arrow). (**E**) Result of the 2D approximation depicting an elevated shear stiffness of the liver of a patient with severe liver fibrosis (F4-grade) [kPa]. (**F**) Corresponding map of the shear viscosity resulting from a full 3D inversion of the wavefield [kPa]. Data from University Hospital Frankfurt am Main, Hesse, Germany.

Figure 5 shows the overarching mathematics and physics governing the 3D wave propagation. Under certain assumptions, it is however possible to simplify the 3D equation, which ultimately necessitates the measurement of the 3D displacement vector within a volume and the calculus of third-order spatial derivatives, which puts high requirements on the SNR of the data. Another approach assumes that the total wavefield is composed of independent plane waves and cuts out in Fourier space a “pie-chart” section in order to virtually recover the individual plane waves (Figure 6A–C) [31]. Additionally, low-pass and high-pass filters are utilized to suppress noise and compressional wave components, respectively. The resulting wave is a quasi-plane shear wave which allows the recovery of local stiffness values without the necessity of measuring all the wave components within a volume (Figure 6D,E). Clearly, this method relies on several assumptions which are not always met. It is nonetheless a rather robust technique which enables quantification of liver stiffness within a single breath hold [37]. However, recovery of the tissue’s viscosity requires the full solution of the 3D wave equation since the previous mathematical operations impact too strongly on the imaginary part of the wave equation to obtain reliable values (Figure 6F).

## 5. MR Elastography Examples Using the Gravitational Transducer

### 5.1. Ultrasound Gel Phantom

**Figure 7:**
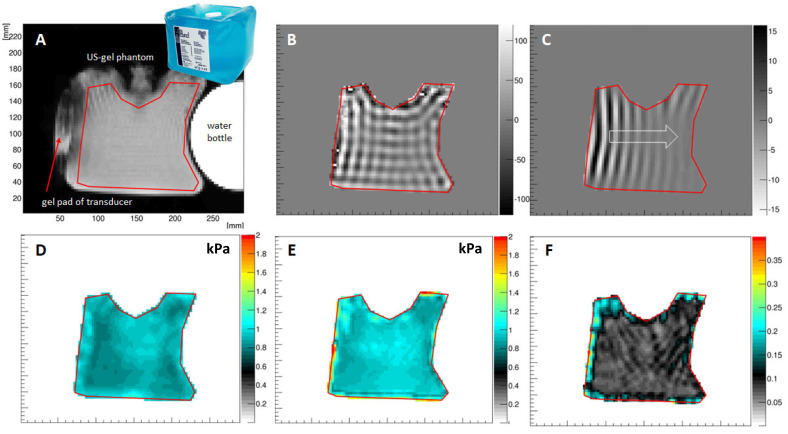
Ultrasound gel phantom results. (**A**) Magnitude image of the experimental setup. Additionally, a water bottle is attached to the US phantom to increase its weight. (**B**) Real part of the displacement field in through-slice direction showing a grid-like pattern which originates from the boundary conditions of the phantom, i.e., its semi-flexible plastic surface. (**C**) One of the plane waves extracted from the directional filter approach presented here is travelling from left to right in the image. (**D**) Magnitude of the complex shear modulus recovered from the 2D approach. The mean value agrees very well with the corresponding gauge obtained via the 3D inversion (**E**). (**F**) Phase angle Y ∈ [0, 1] of the phantom as obtained from the 3D inversion, indicating that the material is exhibiting mainly spring-like properties.

Quality control in MRE is essential to ensure a correct performance of the intricate interplay between MRI data acquisition and mechanical vibration. Figure 7 shows the results from an ultrasound-based phantom (Ultragel Hungary 2000, Budapest, 1211 Hungary) that exhibits a shear modulus of ~0.9 kPa at 60 Hz and has few dispersive properties. The semi-rigid plastic-based surface of the phantom leads to a grid-like pattern of shear waves within the US gel (Figure 7B). The directional filter is capable of extracting the individual plane waves, and Figure 7C shows one of the waves travelling from left to right (arrow). The corresponding 2D inversion yields a proper stiffness of Gd = 0.9 ± 0.1 kPa, which is confirmed by the 3D inversion (Figure 7D,E). The 3D inversion additionally allows quantification of the shear viscosity (Gl), which is very low for such a gel, naturally. A highly homogeneous phase angle (Y) of $Y=0.1=\frac{2}{\pi }atan\left(\frac{Gl}{Gd}\right)$ is retrieved that is consistent with a material that is mostly spring-like with few absorptive properties.

### 5.2. Liver

**Figure 8:**
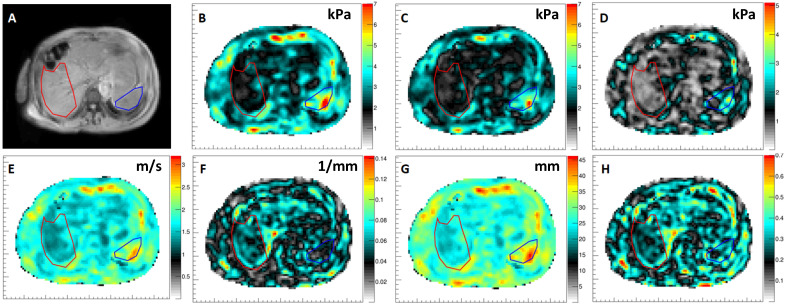
Three-dimensional MRE results in the liver. The outlines of part of the liver and the spleen are highlighted in red and blue respectively. (**A**) Magnitude image of a liver patient with low-grade fibrosis related to non-alcoholic steatohepatitis (NASH). (**B**) Magnitude image of the complex shear modulus |G*| [kPa]. (**C**) Shear elasticity Gd [kPa]. (**D**) Shear viscosity Gl [kPa]. (**E**) Shear speed Cs [m/s]. (**F**) Shear absorption a [1/mm]. (**G**) Shear wavelength l [mm]. (**H**) Shear phase angle Y [0–1]. Data from University Hospital Frankfurt am Main, Hesse, Germany. The institutional ethical review board approved this prospective study. Written informed consent was obtained from the participants.

The mechanical integrity of the liver can be affected by a broad spectrum of diseases, including viruses, drugs, alcohol, or the metabolic syndrome. Non-alcoholic fatty liver disease (NAFLD) is the most common chronic liver disease in Western populations; its prevalence continuously increases due to lifestyle changes, obesity, and type 2 diabetes mellitus [38]. Non-alcoholic steatohepatitis (NASH) is generally considered as the advanced type of NAFLD and is characterized by hepatic inflammation, hepatocellular injury, and fibrogenesis [38]. Lipotoxicity with ongoing inflammation is considered the major pathogenic driver of NASH, aggravating liver injury and promoting liver fibrosis [39]. In approximately 20% of cases, NASH progresses to cirrhosis with increased overall mortality and ultimately the need for liver transplantation [40]. Early identification and initiation of targeted therapy are important to improve patient prognosis. Given the invasive nature of liver biopsy, which carries the risk of numerous complications, MRE emerges as a precise and non-invasive method for evaluating viscoelastic tissue characteristics at first presentation and during follow-up. Due to its ability to evaluate a substantial portion of the liver, MRE can detect focal viscoelastic disparities in all liver segments, in contrast to liver biopsy or transient elastography. Figure 8 shows an example of gravitational 3D MRE applied to a patient with low-grade fibrosis, grounded on NASH. The liver (red ROI in Figure 8B) appears soft with an average value of the magnitude of the complex shear modulus $\left|G^{*}\right|=\sqrt{{Gd}^{2}+{Gl}^{2}}$ of around 2 kPa. Conversely, the spleen (blue ROI in Figure 8B) shows values of around 4.5 kPa, indicating a considerably higher stiffness. This difference is similarly reflected in the elasticity Gd (Figure 8C) as well as in the viscosity (Figure 8D). While both elasticity and viscosity express the biomechanics from the point of view of the material, it is equally possible to express it from the point of view of the propagating wave. Thus, the numbers can be matched to ultrasound elastography when properly considering dispersive effects since the US-based concepts using an acoustic radiation force to generate shear waves operate at a higher central excitation frequency (~150 Hz). Figure 8E shows the corresponding image of the shear wave speed with the wave propagating faster in the spleen than in the liver. Intriguingly, the wave absorption is higher in the liver than in the spleen (Figure 8F). The most simple and robust quantity which can be extracted from the wave equation is the shear wavelength (Figure 8G). As expected, the shear wavelength is short in this patient’s liver, while it is larger in the spleen, reflecting the corresponding soft and stiff material properties. The last biomarker, which carries great potential in quantifying subtle changes in tissue mechanics, is the shear phase angle Y, which reports the ratio of shear viscosity to shear elasticity scaled to the range from [0–1]. A value close to zero indicates mainly an elastic behavior (spring-like) of the material, while a value close to one is indicative of viscous dashpot-like behavior. Clearly, the spleen is exhibiting a higher phase angle, showing that its overall behavior is more dashpot-like than that of the liver. Current research is focusing on expanding hepatic 3D MRE to a wide-bore low-field MR system [41] and exploring which biomarker carries which diagnostic value. We have so far seen a linear behavior of shear speed with the liver fibrosis grade and a nonlinear relationship between viscosity and inflammation [6,25]. Intriguingly, the phase angle Y has shown very little to no dependence on the underlying pathology.

### 5.3. Kidney and Prostate

**Figure 9:**
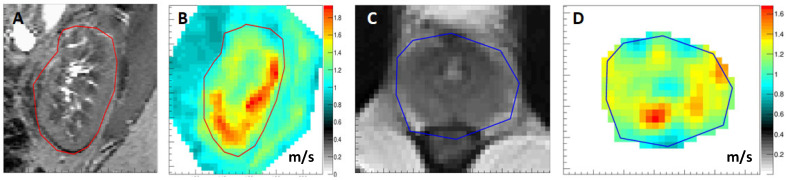
Three-dimensional MRE results in the kidney and the prostate. The outlines of the kidney and the prostate are highlighted in red and blue respectively. (**A**,**B**) T2-weighted anatomical image of the kidney and corresponding image of the shear wave speed [m/s]. Data from University Hospital Vienna, Austria. (**C**,**D**) T2-weighted anatomical image of the prostate and corresponding image of the shear speed [m/s]. Data from University Hospital Frankfurt am Main, Hesse, Germany. The institutional ethical review board approved this prospective study. Written informed consent was obtained from the participants.

Figure 9A,B depict an example of 3D MRE applied to the kidney. The gravitational transducer is located on the posterior lateral abdominal wall of the patient emitting waves in an AP direction [42]. The cortex, medulla, and central liquid zone are all well distinguishable in the corresponding maps of the shear speed. Bear in mind that liquid is extremely soft in terms of shear, while very stiff in terms of compression due to its incompressible nature. Also, the shear wave speed is well aligned with the anatomical image, as the renal sinus reaches into the cranial part of the kidney. Figure 9C,D show an example of the application to the prostate with the transducer strapped against the pubic bone and the patient in a supine position. The urethra, as well as the different zones within the prostate, are well delineated in the speed map.

### 5.4. Breast

**Figure 10:**
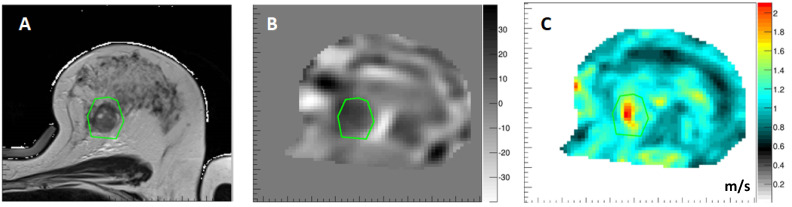
Three-dimensional MRE results in the breast. (**A**) T1 weighted anatomical image of the breast depicting a tumor within the green ROI. (**B**) Corresponding Z-component of the curl field demonstrating that wave propagation is not in a plane wave fashion due to the very complex boundary conditions. (**C**) Resulting shear wave speed image showing the tumor as stiff object within the otherwise rather soft breast tissue. Data from King’s College London, UK. The institutional ethical review board approved this prospective study. Written informed consent was obtained from the participants.

**Figure 11:**
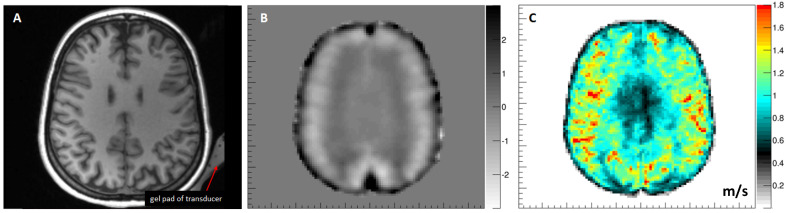
Three-dimensional MRE results in the brain. (**A**) Axial reformation of a T1-weighted 3D MPRAGE structural image of the brain. (**B**) Corresponding image of the Z-component of the curl showing the complex and intricate shear wave pattern within the brain. (**C**) The resulting image of the shear wave speed shows a very high level of symmetry within the brain parenchyma and low values within the lateral ventricles, as expected [m/s]. Data from University Hospital Heidelberg, Germany. The institutional ethical review board approved this prospective study. Written informed consent was obtained from the participants. The data were acquired in one of the investigators in preparation of a study approved by the Ethics Committee of Heidelberg University.

The application of MRE to the breast [43] necessitates adaptation of the gravitation transducer to the anatomical constraints of the breast coil. This was conducted in the context of the EU-funded Horizon2020 project (FORCE), where it was incorporated into the breast MRI biopsy coil. Figure 10 shows results from a breast cancer patient where the presence of the tumor is well depicted within the shear wave speed image. The corresponding Z-component of the curl field shows that wave propagation is complex and is similar to the case of brain MRE (Figure 11); hence, the plane wave assumption for performing 2D reconstruction approaches might be challenged. MRE is currently under investigation in women undergoing neoadjuvant chemotherapy for breast cancer to determine the response or resistance of the tumor early during treatment. Should MRE prove to be a useful biomarker of response or resistance, it will enable the oncologist to switch patients who are not responding to a particular drug regimen of neoadjuvant chemotherapy to an alternative drug regimen or guide the patient to early surgical intervention, minimizing unnecessary toxicity due to an ineffective chemotherapeutic regimen and thereby improving quality of life.

### 5.5. Brain

The application of MRE to the human brain is challenging due to the protective nature of the skull. Figure 2C shows the setup developed at Heidelberg University Hospital with the transducer located within the head coil and transmitting waves in an inclined fashion to generate wave motion in all three directions (Figure 11). The corresponding Z-component of the curl field shows that the shear waves propagate in a rather complex manner through the brain. The resulting shear wave speed image (Figure 11C) depicts a high level of symmetry, as expected from a healthy volunteer’s brain. The subcortical white matter exhibits higher values of shear wave speed than cortical grey matter. The shear wave speed drops within the ventricles since cerebrospinal fluid cannot be sheared and hence exhibits very low values. The non-invasive evaluation of cerebral biomechanics is of high interest and has the potential to improve the diagnosis and monitoring of various brain diseases. An obvious application is neuro-oncology as information on stiffness could not only guide the surgical approach but could also help to characterize the tumor in more depth [8]. To this end, the initial clinical studies demonstrated an association between glioma stiffness and genetic features that are prognostically relevant [44,45]. Further areas of interest include neurodegeneration and dementias or neuroinflammation [46,47].

## 6. Conclusions

The gravitational transducer concept, combined with advanced and fast MRI sequences and robust inversion algorithms, enables non-invasive, high-quality, and versatile assessment of biomechanics, leading to the better diagnosis and staging of several clinical conditions, particularly liver fibrosis and inflammation in NAFLD.

## Data Availability

The data sets generated during and/or analyzed during the current study are not available.
